# Current Development and Research in Traditional Chinese Nonpharmacologic Therapy for Pain

**DOI:** 10.1155/2016/4657572

**Published:** 2016-03-22

**Authors:** Fan-Rong Liang, Jianping Chen, Xin Gao, Richard E. Harris, Bai-Yun Zeng

**Affiliations:** ^1^Chengdu University of Traditional Chinese Medicine, Chengdu, Sichuan 610072, China; ^2^University of Hong Kong, Hong Kong; ^3^University of Pennsylvania, Philadelphia, PA, USA; ^4^University of Michigan, Ann Arbor, MI, USA; ^5^King's College London, London, UK

Pain is a common symptom of disease. According to the Medical Expenditure Panel Survey (MEPS), about 100 million adults in the United States were affected by chronic pain in 2008. The annual cost spent on curing pain was greater than the annual costs of treating heart disease, cancer, and diabetes in 2010. With the rapid development of medicine, there are a lot of approaches and medication for relieving pain used in clinic. However, these therapeutic approaches are not always effective and the clinical outcomes as shown in survival rates are still poor. More importantly, there are lots of side effects about these approaches. Seeking innovative therapeutic approaches to improving the outcome of pain treatment is therefore of timely importance. Complementary and alternative medicine (CAM) has attracted much attention as innovative approaches, especially traditional Chinese nonpharmacologic. Traditional Chinese medicine (TCM) has long history in treating pain with good clinical experiences, and a lot of therapeutic methods without pharmacologic have been used in treating pain so far, including acupuncture (*针灸*), tuina (*推拿*), scrapping (*刮痧*), cupping (*拔罐*), and electroacupuncture (電針). In order to find the scientific evidences and convincing data, an increasing number of scholars and research teams focus on traditional Chinese nonpharmacologic to cure pain. The research area is broad, from use of traditional approaches to approaches which combine traditional Chinese and modern technology, from basic research to clinical trial. Some exciting outcomes and recognized conclusions have emerged. Therefore, giving attention to this issue is very significant as it provides the necessary platform for improving and communicating. It is also a good way for the researchers to understand the development of this specific area and to promote the sustainability of research in this international background. In conclusion, developing strategy of nonpharmacologic therapy for pain relief is an exploring and creative activity. It will not only bring about the discovery of new anticancer drugs with lesser or even no side effects, but also make contributions to the globalization and modernization of traditional Chinese medicine. Meanwhile, the great progress of traditional and modern approaches for pain will be beneficial for patients who suffer from pain of different kinds across the globe.

*Fan-Rong Liang* *Fan-Rong Liang* *Jianping Chen* *Jianping Chen* *Xin Gao* *Xin Gao* *Richard E. Harris* *Richard E. Harris* *Bai-Yun Zeng* *Bai-Yun Zeng*

